# Design and evaluation of a novel approach to invisible electrocardiography (ECG) in sanitary facilities using polymeric electrodes

**DOI:** 10.1038/s41598-021-85697-2

**Published:** 2021-03-18

**Authors:** Aline dos Santos Silva, Hugo Almeida, Hugo Plácido da Silva, António Oliveira

**Affiliations:** 1 FEUP-Faculdade de Engenharia, Universidade do Porto, R. Dr. Roberto Frias, 4200-465 Porto, Portugal; 2 ISCA-Instituto Superior de Contabilidade e Administração, Universidade de Aveiro, R. Associação Humanitária dos Bombeiros Voluntários, 3810-902 Aveiro, Portugal; 3 IT-Instituto de Telecomunicações, IST-Instituto Superior Técnico, Torre Norte-Piso 10, Av. Rovisco Pais, 1049-001 Lisboa, Portugal; 4 OLI-Sistemas Sanitários, S.A., Travessa Do Milão, 3800-235 Aveiro, Portugal

**Keywords:** Health care, Quality of life

## Abstract

Multiple wearable devices for cardiovascular self-monitoring have been proposed over the years, with growing evidence showing their effectiveness in the detection of pathologies that would otherwise be unnoticed through standard routine exams. In particular, Electrocardiography (ECG) has been an important tool for such purpose. However, wearables have known limitations, chief among which are the need for a voluntary action so that the ECG trace can be taken, battery lifetime, and abandonment. To effectively address these, novel solutions are needed, which has recently paved the way for “invisible” (aka “off-the-person”) sensing approaches. In this article we describe the design and experimental evaluation of a system for invisible ECG monitoring at home. For this purpose, a new sensor design was proposed, novel materials have been explored, and a proof-of-concept data collection system was created in the form of a toilet seat, enabling ECG measurements as an extension of the regular use of sanitary facilities, without requiring body-worn devices. In order to evaluate the proposed approach, measurements were performed using our system and a gold standard equipment, involving 10 healthy subjects. For the acquisition of the ECG signals on the toilet seat, polymeric electrodes with different textures were produced and tested. According to the results obtained, some of the textures did not allow the acquisition of signals in all users. However, a pyramidal texture showed the best results in relation to heart rate and ECG waveform morphology. For a texture that has shown 0% signal loss, the mean heart rate difference between the reference and experimental device was − 1.778 ± 4.654 Beats per minute (BPM); in terms of ECG waveform, the best cases present a Pearson correlation coefficient above 0.99.

## Introduction

Given the increase of life expectancy in our society, and with cardiovascular disorders (CVDs) still being the leading cause of death globally^1,2^, new methods of disease monitoring and prevention using modern information and communication technologies are needed^3^. For detection and pre-screening of certain CVDs, Electrocardiography (ECG) has been the established first line exam in a clinical setting. The objective of ECG analysis is to verify if there are any cardiovascular problems as perceived by the electrical conduction patterns of the heart, and is widely used to screen for atrioventricular narrowing or blockage, disorders in the activation sequence, ischemia, infarction, arrhythmias, tachycardia or bradycardias (i.e. when the heart beats too fast or too slowly), just to name a few applications. It is an initial test, in the sense that it points out possible suspicions, which should be confirmed with other tests, but it can still be fundamental for early CVD detection, especially if used in long-term non-invasive monitoring^4^.

Significant work has been made in the domain of wearable devices to bring the ECG to the masses^5^, with examples like the Apple Watch showing competitive results or, in some reported cases, even surpassing standard medical approaches^6–8^. Nevertheless, despite providing new screening opportunities, wearable devices are hindered by challenges such as the need to perform a specific action for measurements to be obtained or users needing to remember wearing them^8^, and still shadowed by the abandonment rates of up to 30%^9^. This is paving the way for “invisibles” or “off-the-person” sensing approaches^10,11^, in which the sensors are integrated in everyday use objects, hence enabling data acquisition as an extension of subjects’ everyday activities.

Regular measurement of health and wellbeing with sensors integrated in the home environment is a topic of growing interest in the international community, as shown by previous work on the topic of “smart homes”^12–14^. However, biomedical sensors such as the ECG have specific requirements in what concerns the interface with the body. Our proposal is to use an approach that non-intrusively monitors the ECG without directly attaching sensors and transducers to the body. To avoid specific procedures to be performed by the users, in this work we present an experimental setup targeted at the automatic monitoring of the daily cardiovascular state of the patient during the use of a sanitary facility. As such, we describe a proof of concept (PoC) system, capable of incorporating ECG in a toilet seat for measurement in a passive way. The problem is addressed in an end-to-end approach, covering aspects ranging from materials for the implementation of the interface between the sensors and the body, sensor specifications, industrial design, basic architecture of the system, and characterization of the feasibility of this approach through experimental evaluation with healthy subjects.

The electrical signal of the heart starts with an electrical impulse generated in the sinus-atrial node (SA) also called sinus node, reason for which the normal rhythm also assumes the designation of sinus rhythm. The ECG signal waveform is characterized by: P, QRS and T waves, which are presented sequentially and with a well-defined interval duration; the R peak is typically the most prominent, and the deflection used to segment a beat.

Our work contributes to further extend the state-of-the-art, mainly by addressing problems related with the electrode materials and texture, and by adopting a sensor design that requires less contact points between the sensor and the body. The remainder of the article is organized as follows. Section 2 describes the background and state-of-the-art. Section 3 details the implementation. Section 4 summarizes the experimental evaluation and results. Finally, Sect. 5 outlines the main conclusions and future work directions.

## Background

Previous work can be found within the state of the art focusing on the measurement of biomedical signals by means of sensors integrated in the sanitation facilities environment. Focusing on ubiquitous health care, Kim et al.^15^ performed an ECG measurement study on the toilet lid using capacitively coupled isolated electrodes. In addition, in this study they addressed the impact of the existence of electrical grounding of the body in relation to the analysis of heart rate variability (HRV).

Also, with the purpose of contributing to preventive health care, Conn et al.^16^ present in their study a toilet seat-based cardiovascular monitoring system with an integrated electrocardiogram, ballistocardiogram, and photoplethysmogram, capable of clinical-grade measurements of systolic and diastolic blood pressure, stroke volume, and peripheral blood oxygenation.

Due to the importance of creating intelligent sanitary equipment, both for measuring physiological parameters and for fall prevention, other studies have been conducted to address these issues. Park et al.^17^ describes easy-to-implement hardware and software for the long-term analysis of a user's excreta through data collection and human health models. The objective is to create a toilet that can perform screening, diagnosis and monitoring and pathologies of specific patient populations.

In order to use this technology for fall detection, Tsuchiyama et al.^18^ suggest an accident detection and patient monitoring system using an ultra-wideband radio sensor system in order to avoid several accidents in the sanitary facility. Huang et al.^19^, pursued the study of a system that measured various physiological parameters, including ECG, body weight and body fat ratio, and that also provided health management function by means of smart toilet seat-mounted electrodes that are used to measure ECG and bioelectric impedance.

These references further reinforce the interest, usefulness and novelty of the topics addressed by our work. In fact, one study suggests that as much as 11% of cardiac arrest episodes are sustained in the toilet^20^. Integrating the sensor in a toilet seat is particularly advantageous, since it is a pervasive object, with which subjects regularly interact, often multiple times throughout the day. The interaction mode is such that it eliminates constraints typically associated with wearable devices (namely the need for a voluntary action from the user prior to obtaining the measurement)^10^.

In the scope of ECG measurement, key characteristics that differentiate our approach are related with the texture and number of measurement terminals that interface with the user's body. Most solutions use metallic inserts and three contact points with the user; in our approach, we explored the use of conductive polymers that can be produced by 3D FDM (fused deposition modelling) printing and a sensor design with virtual reference (hence requiring only two contact points with the user).

## Proposed approach

### Materials overview

A core component of our work is the interface with the body using polymeric materials capable of being used as electrode. Their conductive properties were considered, taking into account that in a first stage the electrodes needed to be produced by FDM, in which parameters like temperature and printing speed affect the conductivity. Three specific types of polymers were identified, namely: Electrifi (Multi3D, LLC, Cary, NC, USA) (resistivity of 0.006 Ω.cm), Proto-Pasta CDP1(ProtoPlant Inc., Vancouver, WA, USA) (resistivity between 30 Ω.cm and 115 Ω.cm), and Black Magic 3D (Graphene Laboratories Inc., Ronkonkoma, NY, USA) conductive graphene filament (resistivity of 0.6 Ω.cm).

The Multi3D material was discarded due to the high cost, and because it presented a pasty consistency that makes the part lose its form very easily, even showing a significant wear in the normal contact with the skin, hence not suitable for our application. The conductive graphene filament was also discarded due to the high cost. On the other hand, Proto-Pasta presented similar mechanical properties to that of PLA (PolyLactic Acid) filaments, therefore being the selected material.

These polymers use as material base PLA, which is added to other composites that enable them to be electrically conductive. The PLA base allows the materials to be used in desktop 3D FDM printing machines, since the materials fulfil the temperature and printing speed requirements. Although the manufacturers indicate a typical resistivity for the materials, this property varies with the format of the part to produce, and with the printing parameters. As the 3D printed parts are the result of material deposition in the plane (X, Y) and stacked in Z, the resistivity measured in the X and Y axis is different from what is measured in Z, thus causing a resistivity dependent of the part geometry, i.e., the materials possess electric anisotropy.

### Electrode texture

**Figure 1:**
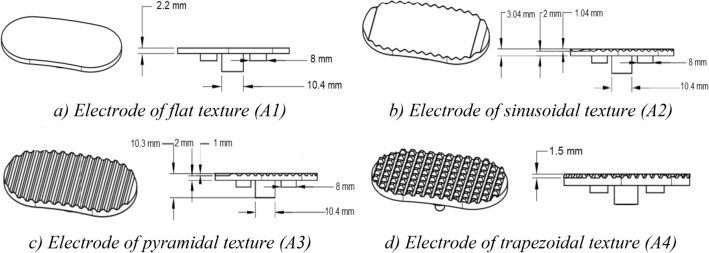
Electrode textures considered for the implementation of our approach.

For ECG data acquisition with the sensors integrated in everyday use objects, an adequate electric contact between the skin of the subject and the electrode can depend on natural barriers such as androgenic hair^1^. This is particularly relevant in the context of our work, in which the sensor is applied on the legs. To address this issue, in our approach we have devised and tested electrodes with different textures to determine which (if any) would have superior performance. We tested a flat/smooth texture, which would be ideal for industrialization, and also sinusoidal, trapezoidal, and pyramidal textures, intended to overcome the androgenic hair barrier. The different textures considered are depicted in Fig. 1.

### Methodology

To test our proposed approach an experimental test bed was created, in which the electrodes were embedded in an off-the-shelve toilet seat (OWCO TAURUS) as four pairs of side pads, for the acquisition of the ECG signal. Each pair of pads matches the textures described in “Electrode texture”, and the electrical connection to the sensor module located at the rear is performed by means of a fused wire (as per the results in “Material resistivity”).

Given that it does not affect the behavior of the sensor and electrodes, the data acquisition system (i.e. from the sensor output onwards), is based on the state-of-the-art BITalino (PLUX, S.A., Lisbon, Portugal) development kit^21^, which has been scientifically validated in previous work^22^. Four ECG sensors with our custom analog front end (one per electrode pair) were used and connected to the analog inputs on the MCU.

With this setup, the toilet seat streams the collected signals via Bluetooth to a receiver (i.e. a computer or mobile phone). The ECG signal is amplified at the analog front end with a gain of 11,000$\times$ and filtered with a band pass filter with passing band [0.5; 40] Hz, limiting the signal band to the typical ECG monitor frequency, reaching the receiver thereafter as raw data. The acquisition is performed with a sampling frequency of 1 kHz and transmitted according to the acquisition protocol^23^; in the acquisition of all four channels, each sample is converted with 10-bit resolution, and packaged in a frame with 7 bytes, resulting in a data rate of 56 kbits/s or 7 Kbyte/s, which is the maximum throughput that the system requires.

**Figure 2:**
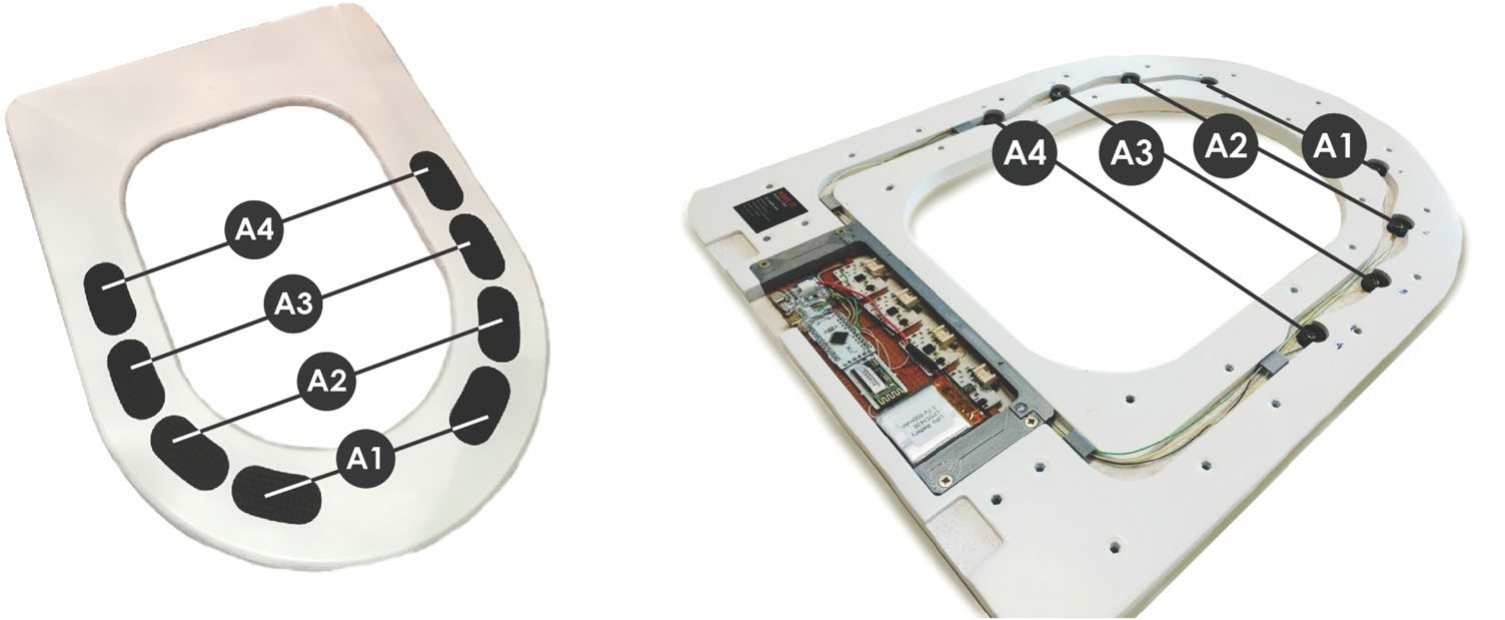
Prototype of the toilet seat, highlighting the electrodes positioning. A1: Flat texture; A2: Sinusoidal texture; A3: Trapezoidal texture; A4: Pyramidal texture.

Figure 2 depicts the experimental setup, complete with the electronics, electrodes and respective wiring. It is also possible to notice the different pads on the left and right sides of the toilet seat top surface, which were always used in the same order/position throughout the tests.

The experimental setup also included an LED and a luminosity sensor to enable the synchronization of the device with external systems; this optical approach was adopted to ensure electrical decoupling between systems. In particular, we use this facility to support simultaneous data collection using the toilet seat and a second system applied to a reference location on the body of the tested subjects (herein considered as the gold standard), in a way that allows the independent ECG time series to be matched in post-processing.

For data analysis and reporting we used Python 3.8, the BioSPPy (0.6.1) library^24^ for the digital filtering and segmentation methods, and PyHRV (0.4.0) for Heart Rate Variability (HRV) analysis. Unless otherwise noted, mean values and standard deviation were computed over all subjects.

## Results

### Material resistivity

To understand the impact of the printing temperature on the material conductivity, several cubic volumes with 1 cm edge were printed, and their resistivity between faces along the Y- & Z-axes was measured. This method is used to determine the volumetric resistivity and, since it does not depend on the volume of the piece, but on its proportions, it is used as a standard measure, with the SI unit, Ω cm. Volumes were produced using three different temperatures. Table 1 summarizes the results of resistivity as a function of temperature. From this part of the work, it was possible to conclude that the temperature that maximizes conductivity for this material is of 230 °C (the maximum limit specified by the manufacturer without compromising stability and reactivity).

**Table 1:** Resistivity on the Y and Z axes as a function of temperature for the material alone (Sample A), fused wire on material (Sample B), and metallic insert embedded on material (Samples C and D).

Temperature (ºC)	Sample A		Sample B		Samples C and D	
	Resistivity (Ω cm)		Resistivity (Ω cm)		Resistivity (Ω cm)	
	Y-axis	Z-axis	Fusion (Y-axis)	Fusion (Z-axis)	Insert (Y-axis)	Insert (Z-axis)
215	510	540	190	230	260	850
220	500	570	260	300	300	880
230	200	230	150	170	190	240

Given that we are using polymeric materials, the electric contact between the electrode and the connecting wire (to interface with the actual sensor) is yet another variable to consider. To address this aspect, we tested two different methods, namely, fusing of the wire in the electrode, and using a metallic insert to which the wire could be screwed. In the latter case, the wire is heated and inserted in the electrode as it is being produced. In the former case, the metallic insert is heated to the point where it melts into the polymer; after that, a brass terminal in the electric wire is screwed to the insert in such a way that the electrode makes contact with metallic insert and with the connecting wire through the screw.

We tested both options to better assess the effect of the electric contact between the printed electrode material and the connecting wire. Table 1 also presents the result of the measurements. Sample A shows the resistance values for the material alone, while in Sample B a wire was fused next to one of the edges and resistance was measured according to axis Y and Z. In Samples C and D inserts were applied perpendicularly to the axis Y and Z, and the resistance was measured in each.

Results show that the resistivity decreases with a higher printing temperature, as specified by the manufacturer, however, the resistivity is much higher than the announced 30 Ω cm along the Y-axis and 115 Ω cm along the Z-axis. Furthermore, this test enables us to conclude that the method of wire fusion presents lower values of resistivity than the method based on metal inserts; the latter finding may be related with the material of the metal inserts (brass—28 IACS (International Annealed Copper Standard)) comparatively to the wire (copper—100 IACS). However, the differences between the two for a temperature of 230 °C are negligible. In the experimental evaluation, both methods were tested, and no significant differences were detected in the quality of the measured data, hence both methods are possible to use in a practical application. Nevertheless, the fused wire requires less components and fewer assembly steps.

### Data acquisition

**Figure 3:**
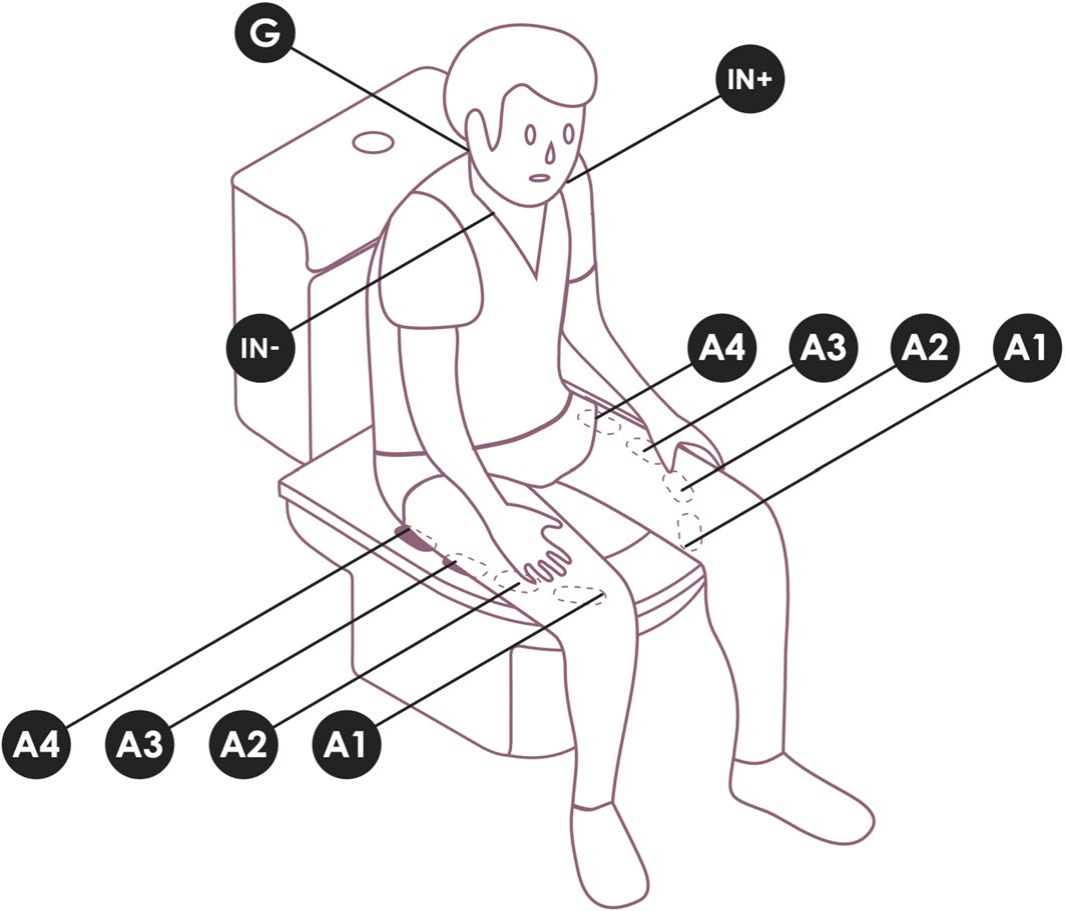
Experimental setup. A1: Flat texture; A2: Sinusoidal texture; A3: Trapezoidal texture; A4: Pyramidal texture*.*

The overall setup used in our study is depicted in Fig. 3. Data was simultaneously collected using our device and the reference system. From our setup, seven data sources are produced, these being in one of the devices O1—LED and A1—ECG REF, while in the other device the ports A1—ECG FLAT, A2—ECG SINUSOIDAL, A3—ECG TRAPEZOIDAL, A4—ECG PYRAMIDAL, and A6—LUMINOSITY SENSOR were used. The ECG REF sensor was applied to the subject with the IN + terminal on the left clavicle, the IN- terminal on the right, and the REF on the cervical—C5/C6 (Fig. 3), corresponding to an Einthoven Lead I. The use of LED and LUMINOSITY SENSOR allows the synchronization of the data collected by both devices in post-processing. In the gold standard (ECG REF sensor) the disposable Covidien (Covidien Ltd., Gosport, UK) KENDALL ARBO H124SG EMG/ECG/EKG surface electrode (24 mm diameter) were used, and while the electrodes described in “Electrode texture” were used on the other sensors. The latter is a dry electrode, while the Covidien electrode is a typical clinical use electrode, which has an adhesive side with non-irritant gel, especially developed to improve conductivity, preventing allergic reactions.

A total of 10 healthy volunteers aged between 26 and 68 years were enrolled; of these participants, 4 were female, 50% had androgenic body hair, and only one had hypertension. For each participant 5 min of data were recorded using the methodology described in “Methodology”, and subjects were asked to have the legs projected to the front, with the heels touching the ground, to ensure a uniform weight distribution across the electrodes. The experimental protocols and methods were followed the guidelines and ethical principles for research involving human subjects set forth by the Declaration of Helsinki, and submitted to and approved by the IT—Instituto de Telecomunicações licensing committee. An informed consent was obtained from all participants.

As described in “Methodology”, the ECG sensor already filters the signal at the hardware level. However, even after this conditioning, the signal may be contaminated by other noise sources; furthermore, for a deeper analysis of the ECG signal trace, segmentation of the heartbeat waveforms is a fundamental step for which the performance is maximized with filtered data. For this, we used the digital filters and segmentation methods included in the BioSPPy; a Finite Impulse Response (FIR) filter of order 300 was used to preserve the signal only in the 3–45 Hz range, while the Hamilton method was used for segmentation^25^.

**Figure 4:**
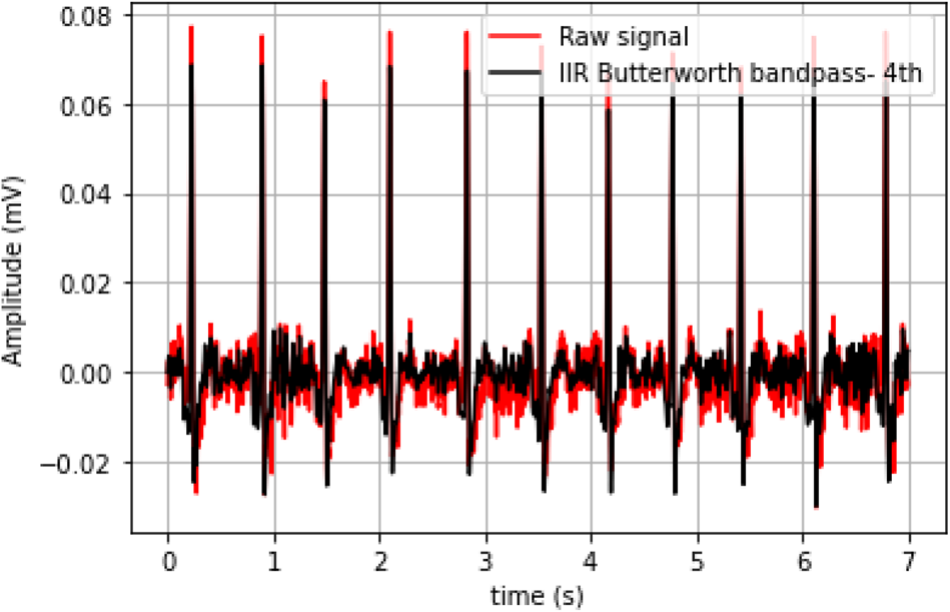
Example of raw and filtered data.

Figure 4 illustrates the original signal, and resulting filtered signal (black trace), in which the elimination of much of the noise present in the source signal (red trace) is already visible. The following “Heart rate” and “Heartbeat waveform morphology”, aim to characterize the system performance in terms of heart rate and ECG signal wave morphology, using the collected data.

Before comparing the experimental signal with the reference, we ensure that only matched segments are used; i.e., as described in “Methodology”, both systems were optically synchronized. In post-processing this enables the temporal alignment of the independent time series. Due to the influence of noise, some QRS complexes of the reference sensor may not have valid matching QRS complexes in the experimental sensor (and vice-versa). For the heart rate analysis we only consider segments in which two or more R peaks are available (allowing the calculation of heart rate in both signals). With this approach, we ensure that the number of QRS segments is the same in both devices. The full waveform morphology (i.e. P-QRS-T waves) is also compared for the segments that have a matching in both the reference and experimental sensors, as further described in “Heartbeat waveform morphology”.

### Heart rate

The goal of this analysis is to characterize potential differences in the heart rate as computed from the signals collected with the reference setup and with our approach; distortions in the R peak may introduce a latency that affects the heart rate calculation, and artefacts in the signal may lead to undetected or wrongly detected peaks. In our work, the segmentation method used is the one described in Ref.^25^. Given that a normal heart rate at rest lies between 60 and 100 BPM*,* when values outside this range are detected we suggest that the analysis is repeated since the values are outside the estimated.

Table 2 shows a summary of the comparative analysis for the heart rate calculation. Each line corresponds to the heart rate derived from the time series from each of the sensors under evaluation, and for which the mean ($\mu$) heart rate is also shown together with the standard deviation ($\sigma$). The heart rate difference between each experimental channel and the reference channel is presented for each pair of matching R peaks between both time series. Finally, the percentage of noisy segments is shown, i.e., corresponding to periods in which the signal is saturated (on the maximum or minimum value), or highly corrupted by noise. Signal Detection Error (SDE in %) is given by the equation:

1 $$SDE\left( \% \right) = \frac{total \,signal - signal \,outside \,the \,measurement \,range}{{total \,signal }}.$$

**Table 2:** Comparative analysis of the heart rate values determined using each sensor.

CHANNEL	Δ#QRS (%)	HR ($\mu \pm \sigma$)	ΔHR ($\mu \pm \sigma$)	SDE (%)	p-value
REF		78.39 ± 8.45			
A1	53.77	82.52 ± 7.71	0.07 ± 6.76	60	0.992
A2	100.10	80.00 ± 5.67	0.15 ± 5.12	10	0.963
A3	113.86	78.72 ± 7.66	− 3.67 ± 5.05	40	0.409
A4	97.15	76.62 ± 8.48	− 1.78 ± 4.65	0	0.644

*Δ#QRS* Percentage of the number of QRS complexes detected by the experimental sensor (Ax, with ${\text{x}} \in \left[ {1, \ldots ,{ }4} \right]$) in relation to the reference QRS (REF), *HR* Heart rate (in BPM), *ΔHR* Difference between HR detected with the (REF) and experimental sensor (Ax), *SDE* Signal Detection Error, *p-value *Two-sided p-value (the unpaired t-test used in the analysis of the data derived from the signals obtained with the A2, A3 and A4 electrodes in relation to the REF electrode).

Based on the results, electrodes A2 and A4 have the best results, by comparison with the gold standard. In order to further characterize the performance, we performed HRV analysis, namely comparing the results of the Detrended Fluctuation Analysis (DFA) and the Poincaré scatter plot. Detrended Fluctuation Analysis (DFA) is a method of non-linear dynamics normally used for the characterization of non-stationary signals. In particular, this technique has been used in HRV to analyze correlations of heartbeat time series intervals. Poincaré scatter graphs, on the other hand, are a geometric visualization technique used to quantify the correlation between two consecutive data points in a time series; this has been used as an indicator of long-term correlation in a HR time series.

The DFA and Poincaré plots were created based on the Normal to Normal (NN) intervals, which are analogous to the R–R intervals but further emphasizes that only R-peaks considered to be normal were considered. From the Poincaré plots we derived the parameters Standard deviation along the minor axis (SD1), Standard deviation along the major axis (SD2), SD1/SD2 ratio, and Poincaré ellipse area (S). The SD1 (Eq. 2) parameter is the standard deviation of the data series along the minor axis and is computed using the time domain Standard deviation of successive differences (SDSD) parameter. The SD2 (Eq. 3) parameter is the standard deviation of the data series along the major axis and is computed using the Standard deviation of successive differences (SDSD) and the Standard deviation of the NN series (SDNN) parameters. The SD1/SD2 ratio is computed as Eq. (4). The area of the ellipse fitted into the Poincaré scatter plot is computed as Eq. (5).

2 $$SD1 = \sqrt {\frac{1}{2}SDSD^{2} } ,$$

3 $$SD2 = \sqrt {2SDNN^{2} - \frac{1}{2}SDSD^{2} } ,$$

4 $$SD_{ratio} = \frac{SD1}{{SD2}},$$

5 $$S = \pi \times SD1 \times SD2 .$$

DFA, much like the Hurst exponent, is used to find long-term statistical dependencies in time series. The idea behind DFA originates from the definition of self-affine processes. A process X is said to be self-affine if the standard deviation,$\delta ,$ of the values within a window of length n changes with the window length factor L in a power law, Eq. (6):

6 $$\delta_{{\left( {X,L \times n} \right)}} = L^{{H \times \delta_{X,n} }} ,$$

where $\delta$(X, k) is the standard deviation of the process X calculated over windows of size k. In this equation, H is called the Hurst parameter, which behaves indeed very similar to the Hurst exponent. Like the Hurst exponent, H can be obtained from a time series by calculating $\delta$ (X,n) for different n and fitting a straight line to the plot of log($\delta$(X,n)) versus log(n). To calculate a single $\delta$ (X,n), the time series is split into windows of equal length n, so that the ith window of this size has the form Eq. (7).

7 $$W\__{{\left( {n,i} \right)}} = \left[ {x_{i} ,x_{i + 1} ,x_{i + 2} , \ldots ,x_{i + n - 1} } \right].$$

The value $\delta$ (X,n) is then obtained by calculating $\delta$ (W_(n,i)) for each i and averaging the obtained values over i.

**Figure 5:**
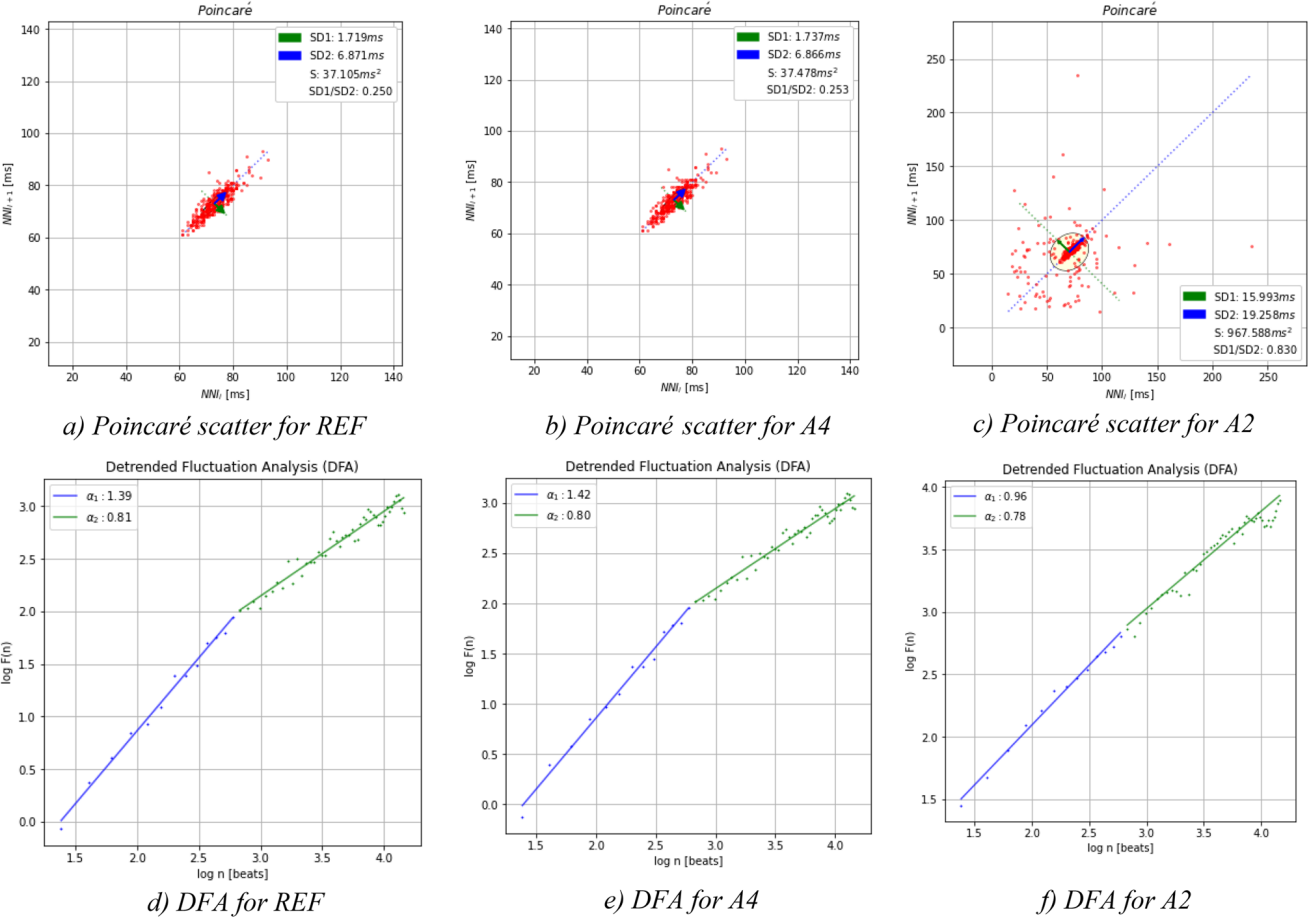
DFA and Poincaré plots for REF, A4, and A2 electrodes.

Figure 5 and Table 3 depicts the DFA and Poincaré data for a randomly selected subject, showing that, while the REF and A4 data exhibit comparable trends, A2 has a higher amount of outlier NN intervals. Based on these findings, we can conclude that the electrode texture A4 (“Electrode texture”) has the best performance, when compared to the gold standard, hence being the data source considered hereinafter for further analysis.

**Table 3:** Comparative analysis of the heart rate values determined using each sensor.

Channel	*Poincaré*				DFA	
	SD1 (ms)	SD2 (ms)	S (ms^2^)	$${\raise0.7ex\hbox{${SD1}$} \!\mathord{\left/ {\vphantom {{SD1} {SD2}}}\right.\kern-\nulldelimiterspace} \!\lower0.7ex\hbox{${SD2}$}}$$	$$\alpha_{1}$$	$$\alpha_{2}$$
REF	1.97 ± 89.40	6.81 ± 47.30	38.39 ± 10.44	0.259 ± 2.34	1.55 ± 0.89	0.89 ± 0.97
A2	27.94 ± 97.20	19.925 ± 63.70	999.62 ± 34.45	0.934 ± 9.65	0.89 ± 1.23	0.91 ± 1.23
A4	2.03 ± 46.90	6.86 ± 88.50	38.03 ± 12.67	0.261 ± 7.13	1.79 ± 0.99	0.79 ± 0.89

### Heartbeat waveform morphology

In addition to the heart rate and HRV analysis, we also performed a heartbeat waveform morphological comparison (i.e., P-QRS-T). For the segmentation of the heartbeat waveforms, the R peaks of the reference ECG are detected first; afterwards, a decision criterion was used for the detection and removal of outlier heartbeat waveforms. In Ref.^25^, two different approaches were proposed, namely DMEAN and DBSCAN, however, previous work has shown that the DMEAN method has better performance comparatively to DBSCAN^21^, reason for which in our study the DMEAN method was used. A heartbeat waveform is considered an outlier, based on the following steps:

1. Calculate the distance $D\left( {x_{i} ,\mu_{i} } \right)$ of each heartbeat to its mean; for this it was necessary to calculate the mean of the templates *(*$\mu_{i}$) and the standard deviation ($\sigma \mu_{i}$). The mean is given by $\mu_{i} = \frac{{\sum x_{i} }}{n}$, being $x_{i}$ the templates and $n$ the total of heartbeat waveforms, and the distance is calculated as $D\left( {x_{i} ,\mu_{i} } \right) = x_{i} - \mu_{i}$.
2. Calculate from $D\left( {x_{i} ,\mu_{i} } \right)$ the mean $\mu_{D} \left( {x_{i} ,\mu_{i} } \right)$ and the standard deviation $\sigma_{D} \left( {x_{i} ,\mu_{i} } \right)$.
3. The condition of the DMEAN method was applied, which is given by the equation: $D\left( {x_{i} ,\mu_{i} } \right) > (\mu D\left( {x_{i} ,\mu_{i} } \right) + 0.5 \times \sigma_{D} \left( {x_{i} ,\mu_{i} } \right)$, comparing all the beats of each heartbeat and at the end presenting a percentage referring to the proportion of false values in relation to the established condition, given by the equation: $\% True Positive \left( {TP} \right) = \frac{{n_{positive} \times 100}}{60}$.

**Figure 6:**
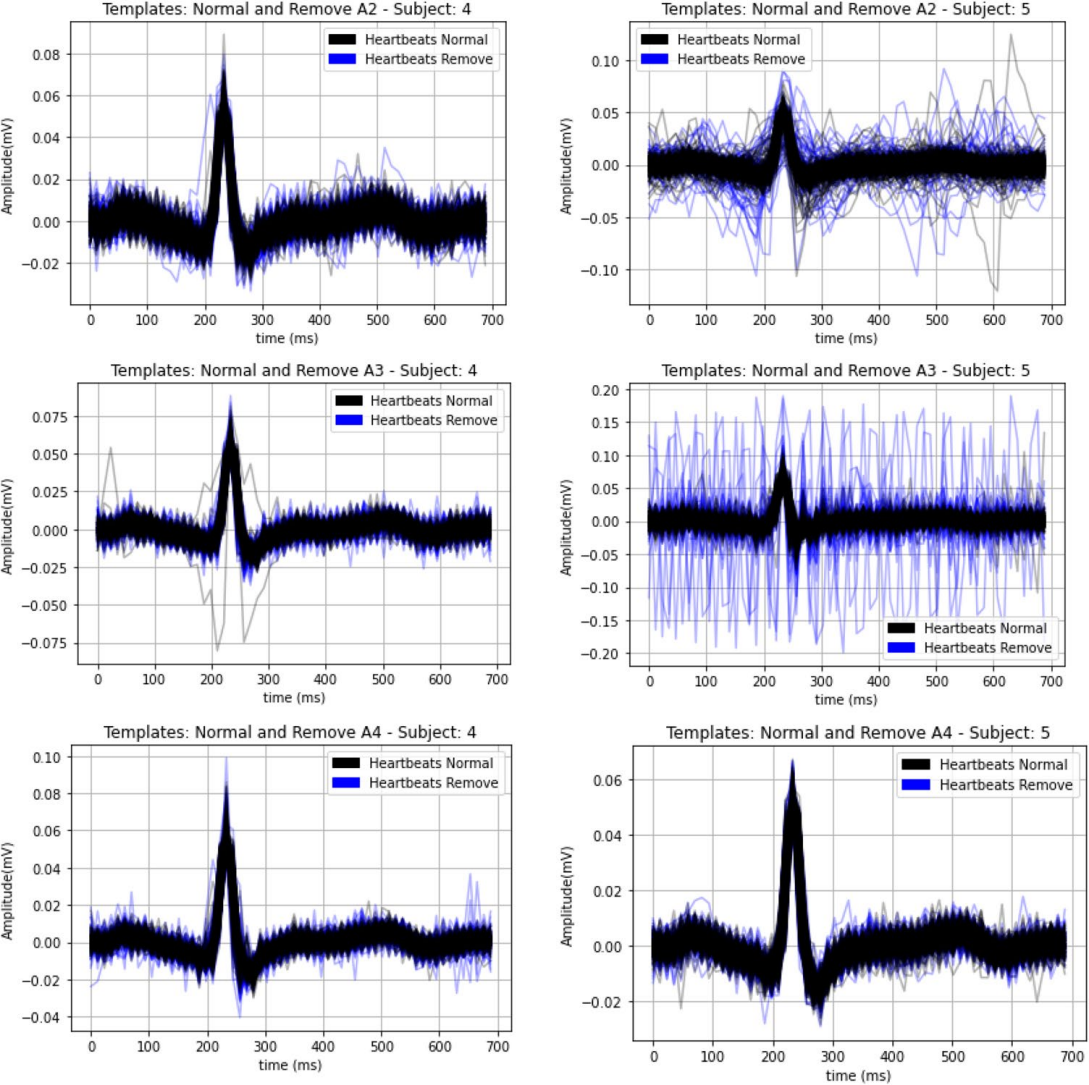
Normal heartbeat waveforms and outliers removed after the decision criteria.

**Figure 7:**
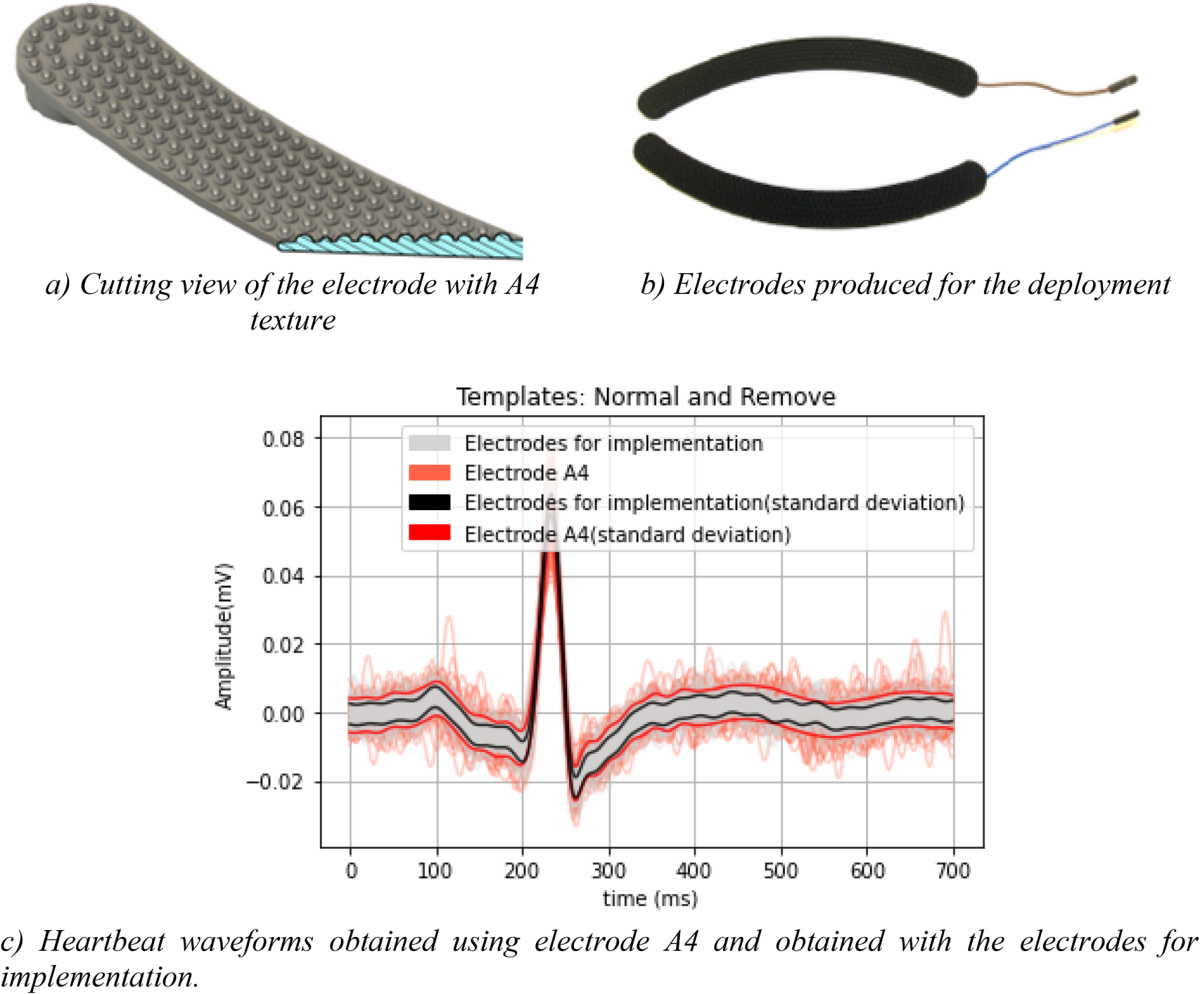
Proposed final electrode texture for deployment and example heartbeat waveform obtained for one participant with the final electrode comparatively with the best performing texture (A4) used in our work (“Electrode texture”).

As an improvement of the DMEAN method, a more specific decision criterion was created for removing outlier heartbeat waveforms. Therefore, after obtaining the percentage of TP, the following decision criterion is established: if the template presents a TP greater than 70%, it will be used, and it is considered to be an outlier otherwise. In Fig. 6 we illustrate the individualized heartbeat waveforms, with the heartbeat waveforms considered valid represented in black and the heartbeat waves considered as outlier represented in blue. Table 4 presents the values of Pearson's correlation coefficient and Normalized Root Mean Square Error (RMSE), for the waveform morphology obtained with the electrodes that demonstrated better performance, calculated according to Eqs. (6), (7), and (8). Based on the results from “Heart rate”, which were further confirmed experimentally, electrode A4 shows the best performance, hence we summarize the results only for this texture. Thus, in Fig. 7 is presented the final texture of the electrode proposed for implantation and an example of the heartbeat wave obtained by it.

8 $${\uprho } = \frac{{\mathop \sum \nolimits_{i = 1}^{n} \left( {x_{i} - \overline{x}} \right)\left( {y_{i} - \overline{y}} \right)}}{{\sqrt {\mathop \sum \nolimits_{i = 1}^{n} \left( {x_{i} - \overline{x}} \right)^{2} } \times \sqrt {\mathop \sum \nolimits_{i = 1}^{n} \left( {y_{i} - \overline{y}} \right)^{2} } }},$$

9 $$\overline{x} = \frac{1}{n}\mathop \sum \limits_{i = 1}^{n} x_{i} ,$$

10 $$\overline{y} = \frac{1}{n}\mathop \sum \limits_{i = 1}^{n} y_{i} .$$

**Table 4:** Pearson correlation coefficient (PCC) and Normalized Root-mean-square error (NRMSE) between the heartbeat waveforms of the reference channel and the experimental channel with the best overall performance (A4).

Subject	A2		A3		A4	
	PCC	NRMSE	PCC	NRMSE	PCC	NRMSE
1	0.56 ± 0.29	28.49 ± 24.97	0.59 ± 0.30	29.03 ± 25.02	0.59 ± 0.30	29.02 ± 24.63
2	0.78 ± 0.30	37.39 ± 10.52	0.87 ± 0.25	34.49 ± 6.53	0.82 ± 0.29	36.16 ± 10.71
3	–	–	–	–	0.36 ± 0.12	57.65 ± 15.22
4	0.98 ± 0.05	24.35 ± 3.98	0.99 ± 0.03	23.94 ± 5.87	0.98 ± 0.05	21.19 ± 4.51
5	0.88 ± 0.15	29.26 ± 11.07	0.98 ± 0.04	31.89 ± 4.58	0.95 ± 0.09	19.86 ± 4.58
6	0.81 ± 0.14	21.59 ± 3.97	–	–	0.83 ± 0.12	18.70 ± 3.48
7	0.95 ± 0.07	11.19 ± 3.01	–	–	0.97 ± 0.04	10.31 ± 2.77
8	0.97 ± 0.09	25.69 ± 16.88	0.98 ± 0.06	25.31 ± 6.42	0.98 ± 0.06	26.16 ± 8.33
9	0.89 ± 0.76	34.07 ± 12.83	0.97 ± 0.98	35.06 ± 3.46	0.99 ± 0.00	32.06 ± 5.46
10	0.99 ± 0.03	31.18 ± 11.89	–	–	0.99 ± 0.02	24.75 ± 5.38

## Conclusions

In this paper, a new sensor design and system for “invisible” ECG has been described and evaluated. Created in the form of a toilet seat, it allows ECG measurements without the use of devices directly attached to the body surface, which brings a new approach to automated pervasive health monitoring systems that work as an extension of people’s everyday life. To acquire the ECG signals on the toilet seat, a specific sensor has been designed, and polymeric dry electrodes with different textures were designed. According to the results obtained, not all textures allow the adequate acquisition of ECG signals, however, the pyramidal texture (A4) showed the best results, as shown by the heart rate, HRV, and morphological analysis of the ECG collected signals. As shown in “Heart rate” and “Heartbeat waveform morphology”, experimental results have confirmed that the flat/smooth texture electrode does not favour an adequate contact with the skin in all subjects. The application of a textured surface presents good results, with the best overall performance being the pyramidal, wherewith the acquisition of the signal was adequately obtained for all subjects.

A prototype of an instrumented toilet seat was created, which aggregates the technical solutions that demonstrated the best performance and format during the design and development process. For final deployment, two elongated electrodes were designed with a shape consistent with the toilet seat lid (Fig. 7b). These retain a texture like a pyramid, but this time with hemispheres, as can be seen in the cut shown in Fig. 7a. The adapted texture is a change of the pyramidal texture in order to improve the comfort and visual perception to the users. Future work will focus on exploring injectable materials with conductive properties and extending the experimental component to the assessment of users with known pathological conditions. In addition, we will study the performance of this approach comparatively to capacitive sensing methods. Nevertheless, this work represents an important prior step to demonstrate the feasibility of ECG data acquisition on the thighs, using polymeric electrodes integrated in a surface with which the subjects normally interact.
